# Effects of reconstruction techniques after proximal gastrectomy: a systematic review and meta-analysis

**DOI:** 10.1186/s12957-020-01936-2

**Published:** 2020-07-16

**Authors:** Zakari Shaibu, Zhihong Chen, Said Abdulrahman Salim Mzee, Acquah Theophilus, Isah Adamu Danbala

**Affiliations:** 1 Department of Gastrointestinal Surgery, Affiliated People’s Hospital of Jiangsu University, Zhenjiang, 212002 Jiangsu People’s Republic of China; 2 Overseas Education College, Jiangsu university, No 301 xuefu road, Zhenjiang, 212013 Jiangsu People’s Republic of China; 3 Department of Gastrointestinal Surgery, Affiliated Hospital of Jiangsu University, Zhenjiang, Jiangsu People’s Republic of China

**Keywords:** Proximal gastrectomy, Double tract reconstruction, Jejunal pouch interposition, Jejunal interposition, Esophagogastrostomy, Double flap, Gastric cancer or neoplasms

## Abstract

**Background:**

Additional studies comparing several reconstruction methods after proximal gastrectomy have been published; of note, it is necessary to update systematic reviews and meta-analysis from the current evidence-based literature.

**Aim:**

To expand the current knowledge on feasibility and safety, and also to analyze postoperative outcomes of several reconstructive techniques after proximal gastrectomy.

**Methods:**

PubMed, Google Scholar, and Medline databases were searched for original studies, and relevant literature published between the years 1966 and 2019 concerning various reconstructive techniques on proximal gastrectomy were selected. The postoperative outcomes and complications of the reconstructive techniques were assessed. Meta-analyses were performed using Rev-Man 5.0. A total of 29 studies investigating postoperative outcomes of double tract reconstruction, jejunal pouch interposition, jejunal interposition, esophagogastrostomy, and double flap reconstruction were finally selected in the quantitative analysis.

**Result:**

Pooled incidences of reflux esophagitis for double tract reconstruction, jejunal pouch interposition, jejunal interposition esophagogastrostomy, and double flap reconstruction were 8.6%, 13.8%, 13.8%, 19.3%, and 8.9% respectively. Meta-analysis showed a decreased length of hospital in the JI group as compared to the JPI group (heterogeneity: Chi^2^ = 1.34, df = 1 (*P* = 0.25); *I*^2^ = 26%, test for overall effect: *Z* = 2.22 (*P* = 0.03). There was also a significant difference between JI and EG in length of hospital stay with heterogeneity: Chi^2^ = 1.40, df = 3 (*P* = 0.71); *I*^2^ = 0%, test for overall effect: *Z* = 5.04 (*P* < 0.00001). Operative time was less in the EG group as compared to the JI group (heterogeneity: Chi^2^ = 31.09, df = 5 (*P* < 0.00001); *I*^2^ = 84%, test for overall effect: *Z* = 32.35 (*P* < 0.00001).

**Conclusion:**

Although current reconstructive techniques present excellent anti-reflux efficacy, the optimal reconstructive method remains to be determined. The double flap reconstruction proved to lower the rate of complication, but the DTR, JI, JPI, and EG groups showed higher incidence of complications in anastomotic leakage, anastomotic stricture, and residual food. In the meta-analysis result, the complications between the JI, JPI, and EG were comparable but the EG group showed to have better postoperative outcomes concerning the operative time, blood loss, and length of hospital stay.

## Background

Gastric malignancy is one of the established malignancies associated with high incidence of mortality worldwide. Recent articles have reported the modern diagnostic and treatment methods on eradication of *Helicobacter pylori* to have a direct link on the decline in gastric cancer [1–3]. Gastric cancer rates have dwindled in diagnosis across the world but lamentably, it remains to be a conundrum in the surgical field [4, 5]. The incidence of upper-third gastric cancer, including early gastric cancers, is increasing in Korea, China, and Japan [6–8]. And therefore, cancer-related death rate for proximal gastric cancer (PGC) is higher compared to cancers at other sites of the stomach [9–11]. Several methods tested the sole therapeutic agent deemed reliable as a medicinal procedure of gastric cancer remains to be surgical resection. Despite the fact that there is no general agreement on a preference of procedures as a remedy for PGC [12], additionally, there is still no consensus on the choice of surgical procedures for PGC.

Two distinct stomach resections by a surgical procedure for proximity gastric cancer include total gastrectomy (TG) and proximal gastrectomy (PG). The type of gastrectomy that is chosen of the two by GI surgeons usually relies upon the tumor’s size, stage, and volume of the remnant in the stomach [13]. Accordingly, various types of reconstructions have been investigated. Japanese gastric cancer treatment guidelines propose 3 types of reconstructions for proximal gastrectomy: esophagogastrostomy, jejunal interposition, and double tract jejunal interposition [14]. Furthermore, jejunal pouch interposition and gastric tube stomach esophagogastrostomy are still perceived as dependable methods [15].

Although total gastrectomy may grant more extended lymph node (LN) dissection, proximal gastrectomy is effective as it is able to provide comparable recurrence and survival rates without compromising the physiological functions of the gastric remnant [9]. Actually, it has been expressed by several authors that proximal gastrectomy is the best appropriate surgical operation of early cancer occurring in the upper part of the stomach, since it has the best durability and safety for patients. Their findings have concluded that, evidence as stated prior, proximal gastrectomy has the same survival rates of patients who received the procedure compared to those who received total gastrectomy, while maintaining the physiologic functions of gastric remnant [16]. Nonetheless, proximal gastrectomy may have its eminence in gastric cancer resections; it also comes with undesirable outcomes as well. There is a high primary concern of its possible postoperative complications which consist of reflux esophagitis, which causes excessive heartburn, chest pain, regurgitation of sourness, and anorexia. These complications can diminish the postoperative quality of lives in gastrectomy patients [17].

It should be noted that multiple methods are possible for reconstruction after proximal gastrectomy. Although other additional reconstruction procedures, such as jejunal interposition (JI) [16], jejunal pouch interposition (JPI) [18], and the double-tract reconstruction (DTR) method, [19] in which some distance is maintained between the esophagus and gastric remnant, are efficient in preventing reflux to some extent, the downside is that these procedures can cause other symptoms that are uncommon with EG, such as obstruction and difficulty in endoscopic surveillance of the gastric remnant after surgery [20]. Alternatively, due to the high presence of reflux esophagitis after simple esophagogastrostomy, it has inspired the advancement of new techniques for reconstruction that purposefully aids to prevent reflux, two of which incorporate jejunal pouch interposition and jejunal interposition [16, 21]. The most common type of reconstruction is esophagogastrostomy. A questionnaire was done by 145 Japanese institutions, and it indicated that esophagogastrostomy is desired after a proximal gastrectomy by approximately 50% of institutions [17]. Kamikawa procedure also known as double flap technique (DFT), which was first reported in 1998, is an anti-reflux procedure during EG after PG. DFT consists of a unique multistep process involving the creation of an H-shaped seromuscular double-flap, fixing the esophagus, and the gastric remnant, as well as anastomosis and closure of the double-flap, all of which are basically carried out by hand-sewn techniques. During this procedure, the distal esophagus and anastomosis are embedded in the submucosal layer of the gastric remnant and covered by the seromuscular double-flap, which is meant to function as a one-way valve to stop reflux [22].

The purpose of this study was to disclose the postoperative outcomes of different reconstruction techniques following a proximal gastrectomy.

## Materials and method

Extraction was independently performed by using specially designed data extraction sheets. After we collected 29 full papers, authors, nationality, study design, publication year, type of surgery, and the number of patients were shown in Table 1. Operation time, blood loss, hospital length of stay, anastomotic leakage, anastomotic stricture, reflux esophagitis, and residual food were all considered as the postoperative outcomes.

**Table 1:** Basic characteristics of included studies

Authors	Nationality	year	Design	Groups	Number of patients
wright et al. [23]	Scotland	1987	RS	JI	30
Kameyama et al. [19]	Japan	2004	RC	JI/JPI	13/59
Senmaru et al. [24]	Japan	1999	RC	JI/JPI	12/12
Isobe et al. [25]	Japan	2014	RC	EG/JI/JPI	66/23/12
kazuhiro et al. [26]	Japan	1998	RC	EG/JI	11/14
Adachi et al. [27]	Japan	1999	RC	JI	16/14
kondoh et al. [28]	Japan	2006	RS	EG	10
Yasuda et al. [29]	Japan	2015	RC	EG/JI	25/21
zhao et al. [21]	China	2014	RS	JI	35
Ahn et al. [30]	Japan	2014	RS	DTR	43
Kim et al. [31]	Korea	2016	RS	DTR	27
Zhang et al. [22]	China	2018	RS	EG	62
Kamitaka et al. [32]	Japan	2017	RS	DTR	10
Nomura et al. [17]	Japan	2019	PS	DTR/JI	15/15
Sugiyama et al. [33]	Japan	2018	RS	DTR	10
Tokunaga et al. [34]	Japan	2008	RS	EG/JI	49/58
Ohashi et al. [35]	Japan	2015	RS	JI	65
Tanaka et al. [32]	Japan	2017	RS	DTR	10
Aburatani et al. [36]	Japan	2017	RS	DTR/EG	19/22
Yang et al. [37]	Korea	2016	RS	DTR	16
Nakmura et al. [38]	Japan	2014	RS	EG/JI/JPI	65/25/12
Hong et al. [39]	China	2015	RS	DTR	21
Katai et al. [14]	Japan	2010	RS	JI	128
Masuzawa [40].	Japan	2013	RS	EG/JI	49/32
Takayama [41].	Japan	2018	RS	JI	32
Kano et al. [42]	Japan	2019	RS	DFR	51
Koruda et al. [20]	Japan	2019	RS	DFR	464
Omori et al. [43]	Japan	2017	RS	DFR	32
Saeki et al. [44]	Japan	2018	RS	DFR	13

### Data extraction

General characteristics of included studies, such as the country, study design, type of reconstruction, publication year, and a number of patients, were shown in Table 1. The reconstruction types were classified into 5 groups: double tract reconstruction, jejunal pouch interposition, jejunal interposition, esophagogastrostomy, and double flap technique. Incidence of postoperative outcomes such as anastomotic strictures, anastomotic leakage, residual food, and reflux esophagitis was confirmed by endoscopic examination. Reflux esophagitis was classified by the Los Angeles classification; degree B or more severe degrees were extracted. In studies reporting incidences of reflux esophagitis during various periods, incidences during the 12th month were considered.

Studies separately reporting each postoperative complication were also selected where the most frequently observed complication was considered. All analysis was based on previously published studies, meaning, no ethical approval and patient consent were required.

### Characteristics of included studies

The characteristics of the 29 studies are listed in Table 1. The published year ranged from 1966 to 2019. The studies included 21 retrospective studies, 1prospective studies, and 6 randomized control. The reconstruction types were classified into 5 groups: double tract reconstruction, jejunal pouch interposition, jejunal interposition, esophagogastrostomy, and double flap technique (Fig. 15 a–e).

#### Inclusion criteria

- Only full published paper in English or translated paper.
- Comparative and non-comparative studies included.
- Open or laparoscopic procedure of proximal gastrectomy.
- Laparoscopic or open techniques

#### Exclusion criteria

- Animals or lab studies excluded.
- Case reports, comments, letters, and reviews without original date excluded.
- Postoperative outcomes are not recorded.
- Total gastrectomy, subtotal gastrectomy, and distal gastrectomy are excluded.

## Statistical analysis

Statistical analysis was performed using the Review Manager (RevMan) software, version 5.0 offered by the Cochrane collaboration. Continuous variables were pooled using the mean difference (MD) with a 95% confidence interval (95% CI), and dichotomous variables were pooled using the odds ratio (OR) with a 95% CI. Random effect and fixed effect models were computed under statistical methods of Mantel-Haenszel (for OR or RR). Heterogeneity among studies was assessed using the inconsistency statistic (*I*). If *I* was < 50%, the eligible studies were considered to be homogenous; hence, the fixed effect model was used. In contrast, if *I* was > 50%, the pooled results were said to be significant, heterogeneous, and the random effect model was used instead.

## Results

### General characteristic of the analyzed patients

**Fig. 1:**
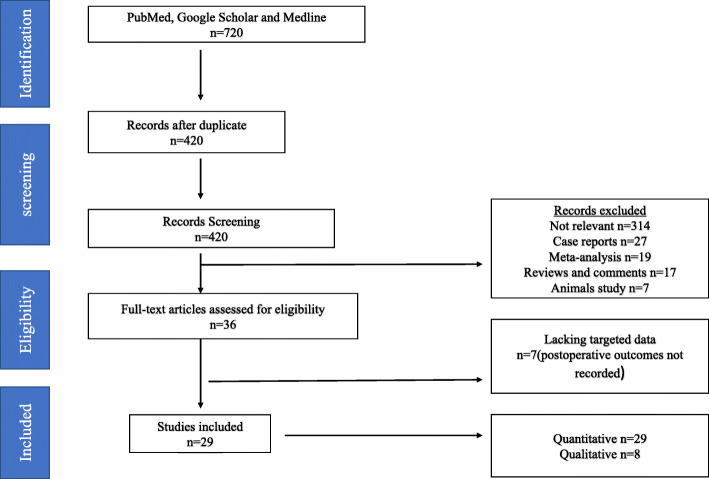
Flow chart of the literature screening, exclusion and inclusion process

A total of 720 studies were searched. Of these searched studies, 420 remaining after duplicates were removed, and 420 articles were screened and resulted in 314 irrelevant, 27 reports, 19 meta-analyses, 17 reviews and comment, and 7 animal studies. A total of 36 full-text articles were carefully studied and 7 studies lacking targeted data. The remaining 29 studies were included as shown in Fig. 1. Of the 29 studies, 29 were analyzed as quantitative studies and 8 out of the 29 for qualitative studies. The 29 studies included in quantitative studies comprised of several reconstructive techniques. Their studies were 3 papers from china, Zhao et al. [39], Zhang et al. [24], and Hong et al. [25], 23 papers from Japan, senmaru et al. [26], kameyama et al. [21], isobe et al. [27], kazuhiro et al. [28], adachi et al. [29], kondoh et al. [30], yasuda et al. [32], ahn et al. [45], nomura et al. [33], sugiyama et al. [46], tokunaga et al. [35], ohashi et al. [47], tanaka et al. [38], aburatani et al .[40], nakamura et al. [41], katai et al. [16], masuzawa et al. [42] and takayama et al. [43], kano et al. [44], koruda et al. [22], omori et al [31], and saeki et al. [37], 2 papers from Korea, Kim et al. [23] and yang et al. [34], and 1 paper from Scotland, wright et al. [36]. Studies reported postoperative outcomes such as reflux esophagitis, anastomotic leakage, anastomotic stricture, and residual food shown in Table 2.

**Table 2:** Qualitative analysis postoperative outcomes

Authors	Reflux esophagitis	Anastomotic stricture	Anastomotic leakage	Residual food
Double tract				
Ahn et al. [30]	2/43 (4.65%)	2/43 (4.65%)	–	21/43 (48.9%)
Kim et al. [31]	2/17 (11.8%)	0/17 (0%)	1/17 (5.9%)	–
Kamitaka et al. [32]	2/10 (20%)	–	0/10 (0%)	–
Nomura et al. [17]	1/15 (6.7%)	1/15 (3.3%)	0/15 (0%)	2/15 (13.3%)
Sugiyama et al. [33]	–	0/10 (0%)	1/10 (10%)	–
Aburatani et al. [47]	2/19 (10.5%)	0	0	–
Yang et al. [37]	0	–	–	–
Tanaka et al. [32]	2/10 (20%)	0	0	0
Hong et al. [39]	0	0	0	–
Total	11/114 (9.6%)	3/85 (3.5%)	2/52 (3.9%)	23/58 (39.6%)
Jejunal pouch interposition				
Senmaru et al. [24]	–	1/12 (8.3%)	0 (0%)	–
Kameyama et al. [19]	6/46 (13.0%)	–	9/46 (15.3%)	23/46 (50.0%)
Isobe et al. [25]	2/12(16.7)	1/12 (8.3%)	1/12 (8.3%)	–
Nakamura et al [38].	–	1/12 (8.3%)	0	11/12 (91.7%)
Total	8/58 (13.8%)	3/36 (8.3%)	10/58 (17.2%)	34/58 (58.6%)
Jejunal interposition				
Wright et al. [23]	2/30 (6.7%)	1/30 (3.3%)	3/30 (10%)	8/30 (26.7%)
Senmaru et al. [24]	–	2/12 (16.7%)	1/12 (8.3%)	–
Kameyama et al. [19]	3/10 (30.0%)	–	0/10 (0.0%)	3/10 (30.0%)
Isobe et al. [25]	3/23 (13.0)	0/23 (0%)	3/23 (13.0)	–
Kazuhiro et al. [26]	0/14 (0%)	9/14 (64.3%)	0/14 (0%)	–
Adachi et al. [27]	0/16 (0%)	1/16 (6.3%)	0/16 (0%)	–
Yasuda et al. [29]	1/23 (5%)	3/21 (14.3%)	2/21 (10%)	17/17 (100%)
Zhao et al. [21]	2/31 (6.5%)	–	0/35 (0%)	–
Nomura et al. [17]	1/15 (6.7%)	1/15 (3.3%)	0/15 (0%)	4/15 (26.7%)
Tokunaga et al. [46]	3/45 (7%)	–	–	–
Nakamura et al. [38]	–	7/22 (31.8%)	1/25 (4%)	7/22 (31.8%)
Takayama et al. [41]	–	1/32 (3.1%)	–	–
Ohashi et al. [35]	22/65 (34%)	–	6/65 (9%)	–
Katai et al. [48]	–	13/128 (10.2%)	1/128 (0.8%)	–
Masuzawa et al. [40]	5/32 (15.6%)	1/32 (3.1%)	0	–
Total	42/304 (13.8%)	39/345 (11.3%)	15/369 (4.1%)	39/94 (41.5%)
Esophagogastrostomy				
Isobe et al. [25]	12/66 (18.2%)	2/66 (3.0%)	1/66 (1.5%)	–
Kazuhiro et al. [26]	4/10 (40%)	2/10 (20%)	2/11 (18.2%)	–
Kondoh et al. [28]	4/10 (40%)	4/10 (40%)	0/10 (0%)	–
Yasuda et al. [29]	1/23 (4.3%)	0/25 (0%)	0/25 (0%)	–
Zhang et al. [22]	9/62 (14.5%)	11/62 (7.1%)	5/62 (8.1%)	–
Tokunaga et al. [46]	3/38 (8%)	–	–	–
Nakamura et al. [38]	–	12/55 (21.8%)	0	12/55 (21.8%)
Masuzawa et al. [40]	9/49 (18.4%)	2/49 (4.1%)	0	
Abutarani et al. [47]	12/22 (54.5%)	6/22 (27.3%)	0	
Total	54/280 (19.3%)	39/299 (13.0%)	8/174 (4.6%)	12/55 (21.8%)
Double flap reconstruction				
Kano et al. [42]	3/51 (5.9%)	4/51 (8%)	0/51 (0%)	2/51 (3.9%)
Koruda et al. [20]	46/464 (10.6%)	26/464 (5.5%)	7/464 (1.5%)	–
Omori et al. [43]	0/32 (0%)	0/32 (0%)	0/32 (0%)	–
Saeki et al. [44]	1/13 (7.7%)	–	1/13 (7.7%)	–
Total	50/560 (8.9%)	30/547 (5.5%)	8/560 (1.4%)	2/51 (3.9%)

The qualitative studies included 8 studies comparing JI VS JPI and JI VS EG after proximal gastrectomy. All the studies included are from japan: Yasuda et al. [32], kameyama et al. [21], isobe et al. [27], and Kazuhiro et al. [28], tokunaga et al., nakamura et al., masuzawa et al., and Senmaru et al. [26]. Studies for analysis included operative time, blood loss and hospital length of stay, reflux esophagitis, anastomotic stricture, and anastomotic leakage as shown in Table 3.

**Table 3:** Quantitative studies between JI, JPI, and EG

Reference	Reflux Esophagitis	Anastomotic stricture	Anastomotic leakage	Infection	Residual food	Operation time	Blood loss	LOS
JI/JPI								
Senmaru et al. [24]	–	2/1	1/0	1/0	–	297.8 + 17.5/282.9 + 18.6	–	38.6 + 3.5/43.5 + 5.9
Kameyama et al. [19]	3/2	–	0/1	–	30.0%/50.0%	–	–	–
Isobe et al. [25]	3/2	0/1	3/1	–	–	251.7 + 43.6/270.2 + 50.0	230.4 + 204.5/333.9 + 354.4	24.1 + 17.7/21.7 + 16.3
Nakamura et al. [38]	11–	7/1	1/0	–	–	281 + 69/311 + 68	393 + 338/402 + 385	–
JI/EG								
Isobe et al. [25]	3/12	2/0	3/1	–	–	251.7 + 43.6/199.0 + 43.1	230.4 + 204.5/176.5 + 144.2	24.1 + 17.7/15.6 + 10.4
Kazuhiro et al. [26]	0/4	–	0/2	2/0	–	241.2 + 10.2/186.9 + 11.7	424..6 + 64.1/324.0 + 61.4	35.0 + 3.9/28.4 + 4.3
Yasuda et al. [29]	1/1	3/0	2/0	2/3	–	268.8 + 59.6/286.4 + 54.3	307.4 + 264.8/299.2 + 334.5	29.4 + 19.8/18.6 + 3.6
Tokunaga et al. [34]	3/3	–	–	–	–	267 + 11/203 + 9	287 + 32/252 + 50	–
Nakamura et al. [38]	11/12	7/12	1/0	–	–	281 + 69/198 + 25	393 + 338/179 + 158	–
Masuzawa et al. [40]	5/9	1/2	0/0	–	–	230 + 43/185 + 48	331 + 182/280 + 247	23 + 31/20 + 17

### Results of qualitative studies

#### Double tract reconstruction

Eight studies [19, 23, 25, 34, 38, 45, 46, 48] reported double tract reconstruction with a total 171 patients involved. Postoperative outcomes such as reflux esophagitis, anastomotic stricture, anastomotic leakage, and residual food were observed having 9.6%, 3.5%, 3.9%, and 39.6% respectively. Reflux esophagitis and residual food were the most frequently observed as shown in Table 2.

#### Jejunal pouch interposition

A total of four studies [21, 26, 27, 41] concentrated on the postoperative condition of patients who underwent a jejunal pouch interposition with a total of 95 patients involved. Incidences of patients who developed postoperative outcomes such as reflux esophagitis, anastomotic stricture, anastomotic leakage, and residual food were 13.8%, 8.3%, 17.2%, and 58.6% respectively. Residual food and anastomotic leakage were the most observed. The incidence of residual food was reported to be high (Table 2).

#### Jejunal interposition

A total of fifteen studies [19, 21, 26–29, 32, 35, 36, 39, 41–43, 47] reported postoperative conditions of the jejunal interposition with 519 patients that were involved. Incidence of postoperative outcomes was 13.8%, 11.3%, 4.1%, and 41.5% for reflux esophagitis, anastomotic stricture, anastomotic leakage, and residual food respectively. Reflux esophagitis and residual food were the most observed. Residual food had a high incidence (Table 2).

#### Esophagogastrostomy

A total of nine studies [24, 27, 28, 30, 32, 35, 41, 42, 48] reported on esophagogastrostomy. Analysis of the results included reflux esophagitis 19.3%, anastomotic stricture 13.0%, anastomotic leakage 4.6%, and residual food 21.8%. These calculated values show that reflux esophagitis and anastomotic leakage were the most frequently observed postoperative outcomes (Table 2).

#### Double flap technique

Four studies [22, 31, 37, 44] reported double flap reconstruction with a total of 560 patients involved. Postoperative results including reflux esophagitis, anastomotic stricture, anastomotic leakage, and residual food were observed with an incidence of 8.9%, 5.5%, 1.4%, and 3.9% respectively. The occurrence of reflux esophagitis and anastomotic stricture was the most frequently observed complication (Table 2).

### Meta-analysis result for JI versus JPI

#### Reflux esophagitis

**Fig. 2:**
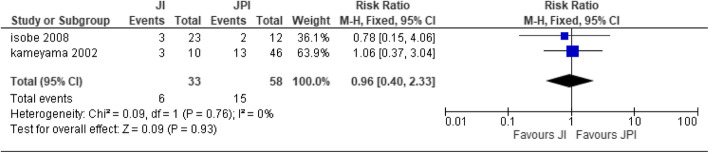
Forest plot of reflux esophagitis

Two papers [21, 27] were used for the analysis with JI (*n* = 33) patients and JPI (*n* = 58) patients. The two studies were homogenous; hence, fixed effect was implemented [heterogeneity: Chi^2^ = 0.09, df = 1 (*P* = 0.76); *I*^2^ = 0%]. There was no statistical significance difference between the two types of reconstructive technique (test for overall effect: *Z* = 0.09 (*P* = 0.93)) (Fig. 2).

#### Anastomotic leakage

**Fig. 3:**
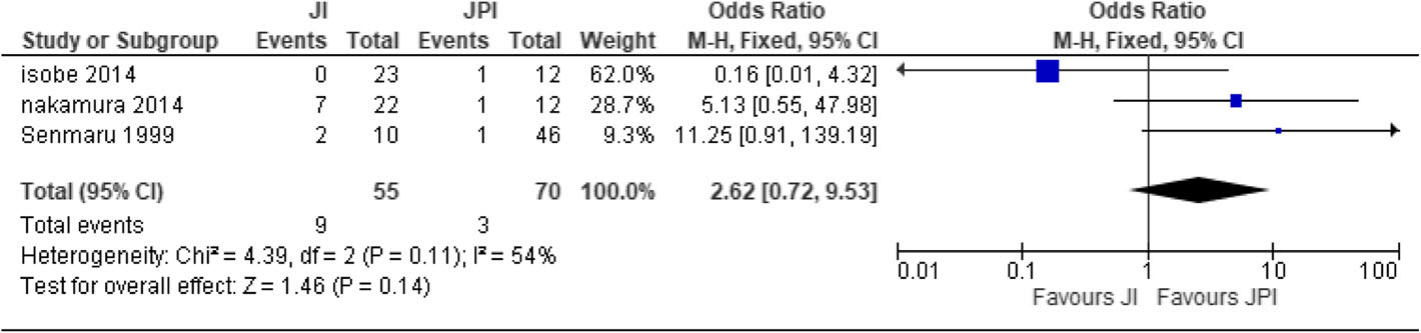
Forest plot of anastomotic leakage

Three studies [26, 27, 41] were used for the analysis with JI (*n* = 55) participants and JPI (*n* = 70) participants. The two studies were moderately heterogeneous; therefore, random effect was used [heterogeneity: Chi^2^ = 4.39, df = 2 (*P* = 0.11); I^2^ = 54%]. There was no statistical significance difference between JI and JPI (test for overall effect: Z = 1.46 (P = 0.14)) (Fig. 3).

#### Anastomotic stricture

**Fig. 4:**
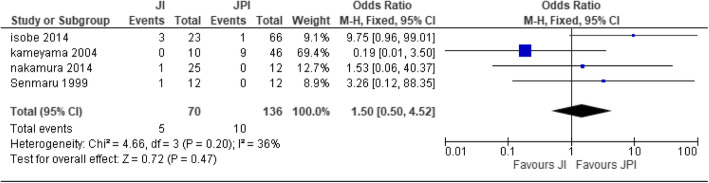
Forest plot of anastomotic stricture

Four papers [21, 26, 27, 41] were used for the analysis of JI (*n* = 70) patients and JPI (*n* = 136) patients. Two studies were homogenous; hence, fixed effect was used [heterogeneity: Chi^2^ = 4.66, df = 3 (*P* = 0.20); I^2^ = 36%]. There was no statistical significance difference between the two methods of reconstruction (test for overall effect: Z = 0.72 (*P* = 0.47)) (Fig. 4).

#### Operative time

**Fig. 5:**
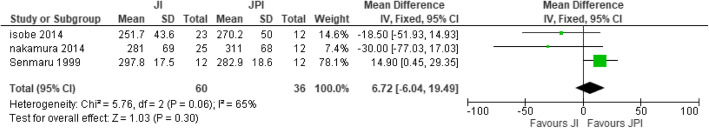
Forest plot of mean difference in operative time

Three studies [26, 27, 41] recorded operative time with JI (*n* = 60) patients and JPI (*n* = 36) patients. There was no significant difference between JI and JPI observed. Random effect model was used [Heterogeneity: Chi^2^ = 5.76, df = 2 (*P* = 0.06); *I*^2^ = 65%] (test for overall effect: *Z* = 1.03 (*P* = 0.30)) (Fig. 5).

#### Blood loss

**Fig. 6:**
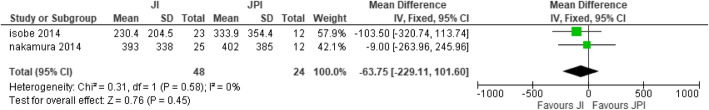
Forest plot of mean difference blood loss

Two studies [27, 41] were recorded for blood loss with JI (*n* = 48) patients and JPI (*n* = 24) patients. No significant difference was reported. Absolute homogeneity was observed; hence, fixed effect model was used [heterogeneity: Chi^2^ = 0.31, df = 1 (*P* = 0.58); *I*^2^ = 0%] (test for overall effect: *Z* = 0.76 (*P* = 0.45)) (Fig. 6).

#### Hospital length of stay

**Fig. 7:**
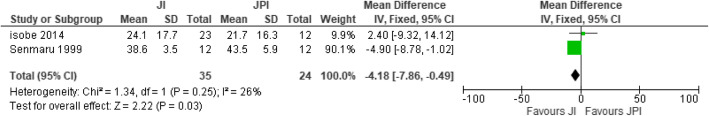
Forest plot of mean difference hospital length of stay

Two studie s[26, 27] recorded length of hospital stay, with JI (*n* = 35) patients and JPI (*n* = 24) patients. There was a significant difference between the two groups. Patients in the JI group had a decreased length of hospital stay as compared to the JPI group. Heterogeneity was absent; hence, fixed effect model was used. [heterogeneity: Chi^2^ = 1.34, df = 1 (*P* = 0.25); I^2^ = 26%.] (test for overall effect: Z = 2.22 (*P* = 0.03)) (Fig. 7).

### Meta-analysis result for EG versus JI

#### Reflux esophagitis

**Fig. 8:**
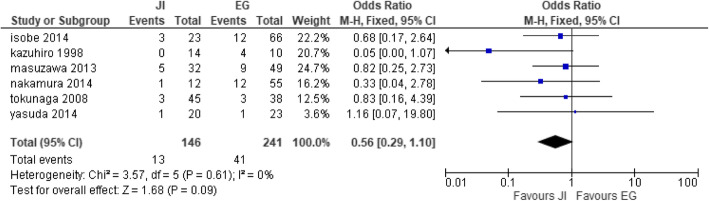
Forest plot of anastomotic leakage

Six studies [27, 28, 32, 35, 41, 42] were collected for reflux esophagitis with JI (n = 146) participants and EG (n = 241) participants. There was no significant difference between the two groups. Homogeneity was observed between the six studies; thus, fixed effect model was used (heterogeneity: Chi^2^ = 3.57, df = 5 (*P* = 0.61); I^2^ = 0% (test for overall effect: Z = 1.68 (*P* = 0.09)) (Fig. 8).

#### Anastomotic stricture

**Fig. 9:**
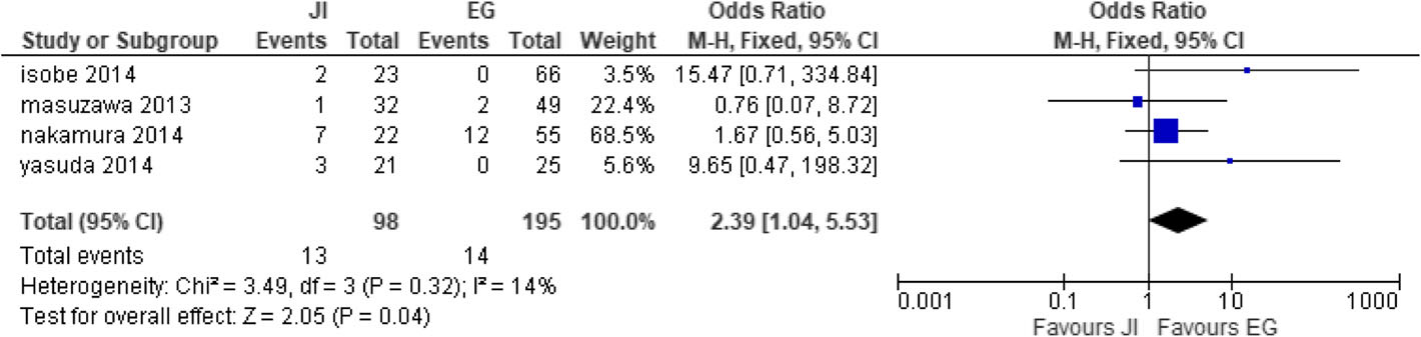
Forest plot of anastomotic stricture

Four studies [27, 32, 41, 42] recorded anastomotic stricture with JI (*n* = 98) participants and EG (*n* = 195) participants. There was no statistically significant difference between the two groups. Homogeneity was observed; hence, fixed effect was used (heterogeneity: Chi^2^ = 3.49, df = 3 (*P* = 0.32); I^2^ = 14% (test for overall effect: Z = 2.05 (*P* = 0.04)) (Fig. 9).

#### Anastomotic leakage

**Fig. 10:**
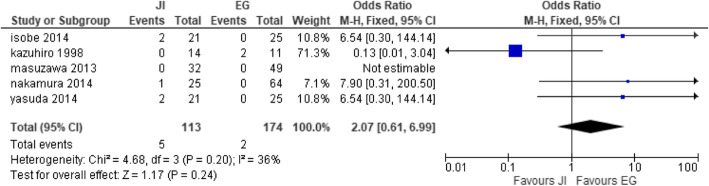
Forest t plot of anastomotic leakage

Five studies [27, 28, 32, 41, 42] reported anastomotic leakage with JI (*n* = 113) and EG (*n* = 174). There was no significant difference between the two groups. Homogeneity was observed [heterogeneity: Chi^2^ = 4.68, df = 3 (*P* = 0.20); I^2^ = 36%] (test for overall effect: Z = 1.17 (*P* = 0.24) (Fig. 10).

#### Operative time

**Fig. 11:**
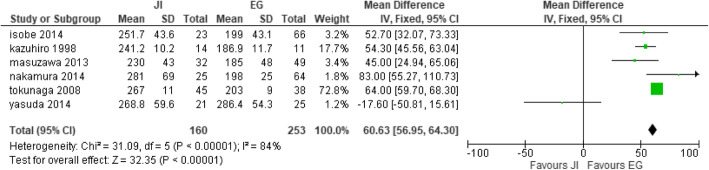
Forest plot of operative time

Six studies [27, 28, 32, 41, 42, 49] collected operative time with JI (*n* = 160) and EG (*n* = 253). A significant difference between the two groups was noted. This study was deemed heterogeneous, as heterogeneity: Chi^2^ = 31.09, df = 5 (*P* < 0.00001); I^2^ = 84%. Thence, a random effect model was used (test for overall effect: Z = 32.35 (*P* < 0.00001). These studies suggested that the EG group had a shorter intraoperative period compared to the JI group (Fig. 11).

#### Blood loss

**Fig. 12:**
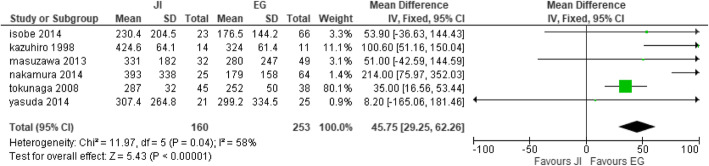
Forest plot of mean difference blood loss

Six studies [27, 28, 32, 35, 41, 42] reported blood loss with JI (n = 160) participants and EG (n = 253) participants. The studies showed a significant difference between the two groups. The EG group reported a decreased amount of blood loss compared to the JI group. Heterogeneity was observed; therefore, fixed effect model was selected (heterogeneity: Chi^2^ = 11.97, df = 5 (*P* = 0.04); I^2^ = 58%, test for overall effect: Z = 5.43 (*P* < 0.00001) (Fig. 12).

#### Length of hospital stay

**Fig. 13:**
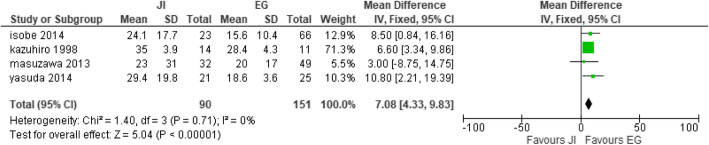
Forest plot of hospital length of stay.

Four studies [27, 28, 32, 42] collected length of hospital stay with JI (*n* = 90) participants and EG (*n* = 151) participants. There was significant difference between the two groups. The EG group showed a decreased length of hospital stay compared to the JI group. Absolute heterogeneity was estimated; hence, fixed effect was selected (heterogeneity: Chi^2^ = 1.40, df = 3 (*P* = 0.71); *I*^2^ = 0%, test for overall effect: *Z* = 5.04 (*P* < 0.00001) (Fig. 13).

## Publication bias

**Fig. 14:**
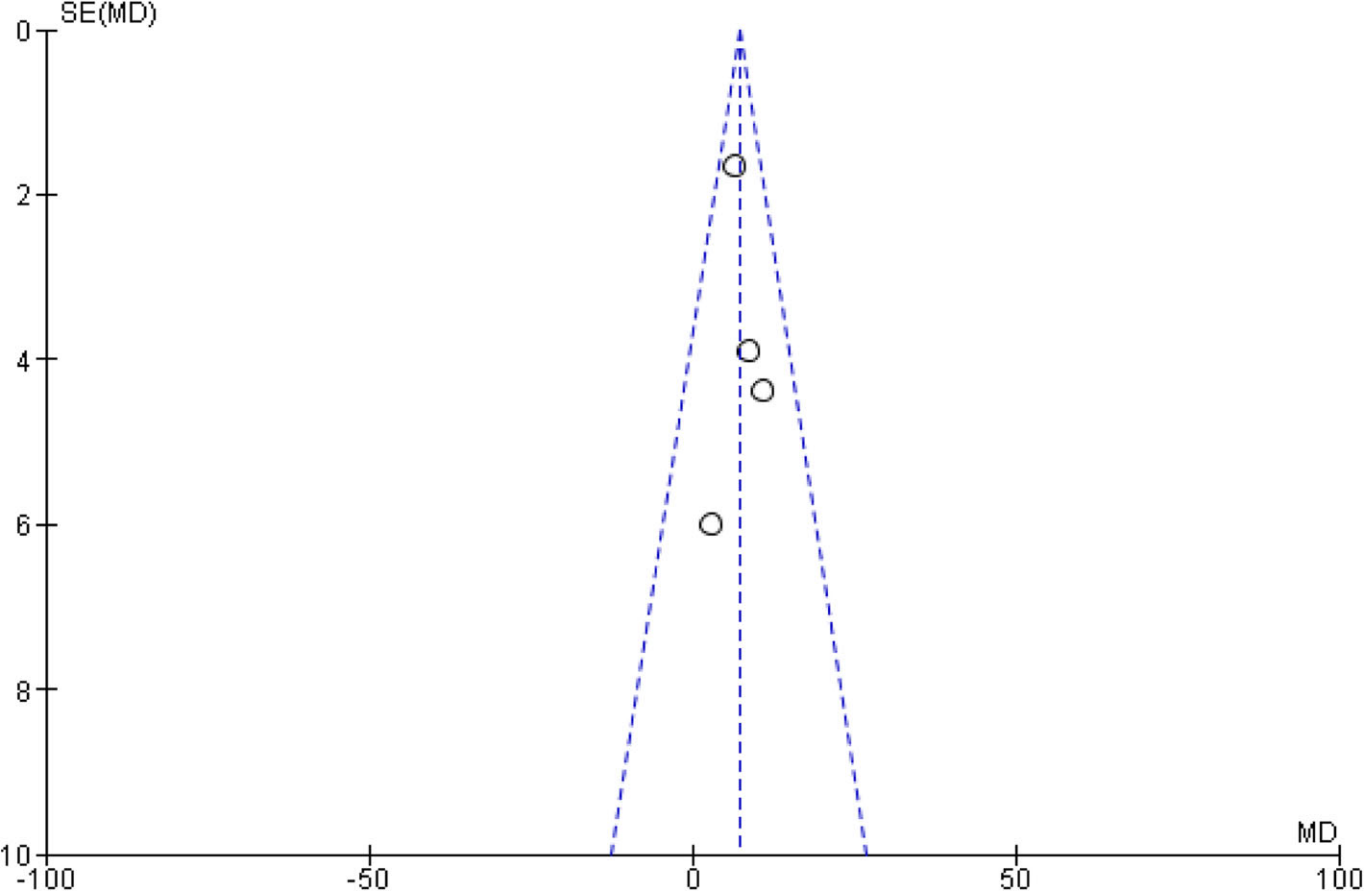
Funnel plot of hospital length of stay for JI and EG

**Fig. 15:**
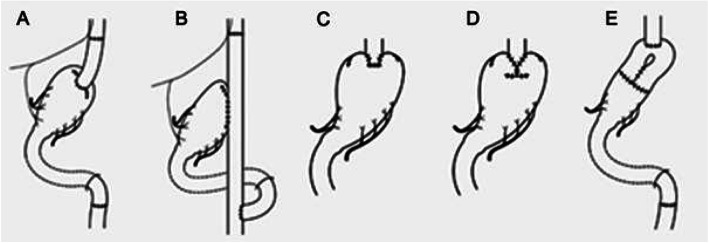
Reconstruction methods after proximal gastrectomy. **a** Jejunal interposition. **b** Double tract method. **c** Esophagogastrostomy. **d** Double flap technique. **e** Jejunal pouch interposition

The funnel plot on the hospital length of stay between EG and JI is shown in Fig. 14. Because all studies laid inside the 95% CI limits, no evidence of publication bias was noted. Egger test was performed to provide statistical evidence regarding funnel plot symmetry. Result still did not reveal any evidence of publication bias (heterogeneity: Chi^2^ = 1.40, df = 3 (*P* = 0.71); *I*^2^ = 0%) (Fig. 15).

## Discussion

The standard surgery preferred for advanced proximal gastric cancer has always favored total gastrectomy, whereas for early-stage proximal gastric cancer, proximal gastrectomy has been the typical surgical solution [29, 50–54]. The study reviewed and analyzed the postoperative outcomes and effect among 29 studies, and some studies were also used for the meta-analysis. Proximal gastrectomy is a considerable resection procedure for an early stage of proximal gastric cancer providing that a sufficient distal resection margin can be ensured. This has been generally accepted by most GI surgeons [55]. However, in the cases of advanced diseases, it has not reached a consensus for its preference. Moreover, if the rate of reflux esophagitis and anastomotic stricture after proximal gastrectomy can be reduced to that of total gastrectomy, proximal gastrectomy may become an advantageous treatment of choice for proximal EGC [45].

Our review and meta-analysis is based on 5 reconstructive techniques. The totality of the anti-reflux reconstruction methods tested demonstrated excellent ability in preventing reflux. The postoperative effects that were under observance included reflux esophagitis, anastomotic stricture, anastomotic leakage, residual food, operative time, blood loss, and length of hospital stay. Additionally, it was found that the preventive reflux methods within several techniques increased the incidence of residual food, reflux esophagitis, anastomotic stricture, and anastomotic leakage in all the reconstruction methods, but DFT recorded in the studies observed low incidence of complications 8.9%, 5.5%, 1.4% and 3.9% respectively. Individual studies also recorded a low incidence of complications [22, 31, 37, 40, 44]. DFT conducted after proximal gastrectomy seemed to be proven effective to lower the outcome as compared to the procedures of DTR, JPI, JI, and EG. Because of the increased anastomosis numbers and technique complexity, postoperative complications, such as anastomotic leakage, anastomosis stricture, and residual food were shown to increase accordingly. Anastomotic stricture (13.0%) was frequently seen in the EG group, anastomotic leakage (18.2%) was observed in esophagogastrostomy, and residual food (100%) were frequently observed in the jejunal interposition group [32] which were in higher incidence. Other studies have also reported stronger anti-reflux efficacy [29, 31, 56], whereas others showed a negative result. The operative time in our study was shorter in the EG group (heterogeneity: Chi^2^ = 31.09, df = 5 (*P* < 0.00001); *I*^2^ = 84%), blood loss was less in the EG group (heterogeneity: Chi^2^ = 11.97, df = 5 (*P* = 0.04); *I*^2^ = 58%), and also, there was a decreased length of hospital stay in the EG group (heterogeneity: Chi^2^ = 1.40, df = 3 (*P* = 0.71); *I*^2^ = 0%); these results could be attributed to the fact that most surgeries were performed laparoscopically [19, 23, 32, 45, 46].

With the development of clinical research, proximal gastrectomy has been gradually replacing the practice of total gastrectomy in treating early gastric cancer located in the upper third of the stomach. Proximal gastrectomy has maintained comparable oncological radicality to the total gastrectomy and the reservoir capacity of the stomach [10, 18] pT1-2 gastric cancer located in the upper third of the stomach has rarely shown any pathological lymph node metastasis at stations #4d, #5, and #6. Although no difference in the long-term survival has been detected between the total and the proximal gastrectomy [49], cardio-esophageal resection and the reserved stomach were shown to significantly increase the risk of gastroesophageal reflux and significantly decrease the postoperative quality of life [18]. PG has a high risk of postoperative gastroesophageal reflux and food stagnation, both of which remarkably decreases the quality of life of patients. Laparoscopic surgery has emerged as an option for the surgical treatment of EGC, including PG. Laparoscopic reconstruction and the improvements of reconstruction methods in PG to prevent regurgitation of gastric contents and to facilitate their discharge into the duodenum are important issues [32]. Concerning EG reconstruction, Adachi et al. [29] and Shiraishi et al .[57] performed EG with a narrow gastric tube and reported it to be a safe and simple procedure with benefits such as shorter operation time, faster recovery, and lower hospital expenses compared with JI and an equally low incidence of reflux esophagitis despite an end-to-end anastomosis. However, regurgitation when lying down cannot be avoided. Meanwhile, Ichikawa et al. [58] reported that EG was performed in an end-to-side fashion using a narrow gastric tube laparoscopically, resulting in favorable clinical outcomes, along with a low incidence of reflux esophagitis and preservation of the physiological function of the remnant stomach. However, the length of the newly created pseudo-fornix was less than 3 cm, which seemed to be inadequate to function as an angle of his for the prevention of reflux. Related research has also confirmed that PGJI improves reflux esophagitis, compared with esophagogastrostomy [16, 35].

Limitations of the techniques were shown to be the incurability of tumors located in the greater curvature and the decreased volume of the stomach, which may alter food intake and nutrition status [29]. So, actual treatment efficacy remains to be determined. There are two major limitations in the present study that was recognized. First, most of the studies on the outcomes of reconstructions for proximal gastrectomy were retrospective case series and non-randomized comparative studies. Also, a downside was that comparisons between the reconstructions were unavailable. Second, in the included studies, various types of complications and various diagnostic criteria of postoperative reflux esophagitis were adopted. As a result, postoperative complications, including esophageal reflux, were described in general; each type of complication was not described in detail. Given the preliminary stage of this study on the reconstruction following a proximal gastrectomy, it was difficult to estimate the occurrence of each complication and to summarize the presence of reflux esophagitis by each diagnostic criterion. It is important that we must first investigate the general data of the reconstructions.

## Conclusion

In summary, anti-reflux reconstruction methods involved in the studies increased the possible rate of surgical complexity by showing a higher incidence of reflux esophagitis, anastomosis stricture, anastomosis leakage, and residual food. Nevertheless, the double flap technique effectively did decrease the risk of complications after proximal gastrectomy. Also, operative time, blood loss, and hospital length of stay were decreased in the EG group because most surgeries were done laparoscopically. Due to the lack of large randomized studies, optimal anti-reflux methods remain to be unidentified.

## Data Availability

The studies included were retrieved from PubMed, Google scholar, and Medline.

## Abbreviations

DTR: Double tract reconstruction
JPI: Jejunal pouch interposition
JI: Jejunal interposition
EG: Esophagogastrostomy
DFT: Double flap technique
EGC: Early gastric cancer
PG: Proximal gastrectomy
PGC: Proximal gastrectomy
TG: Total gastrectomy
WMD: Weighted mean difference
I^2^: *I* square statistic
